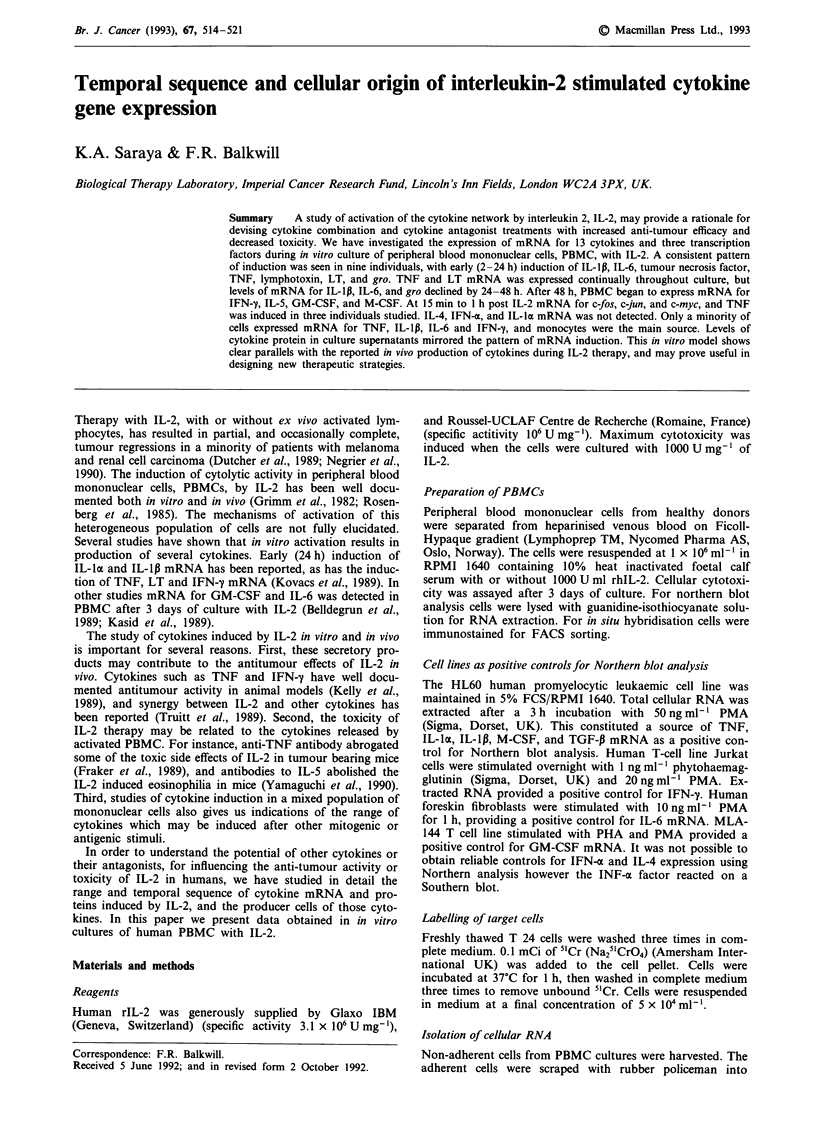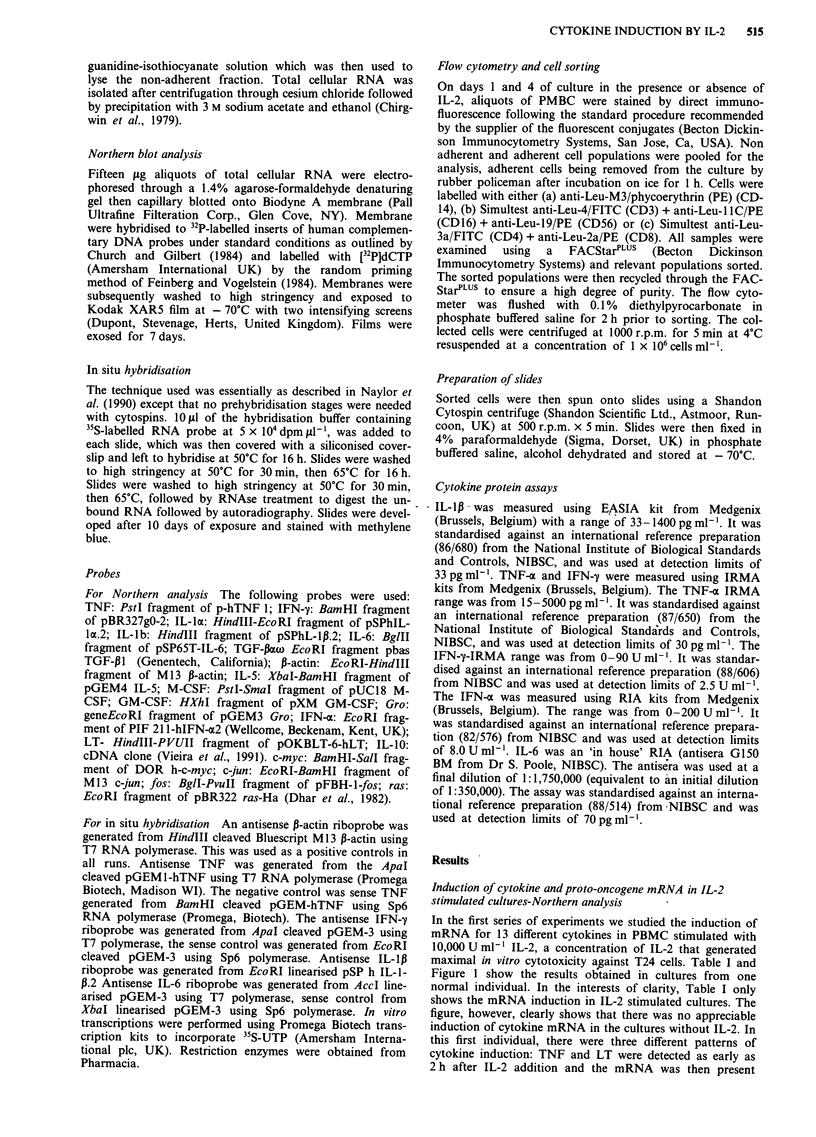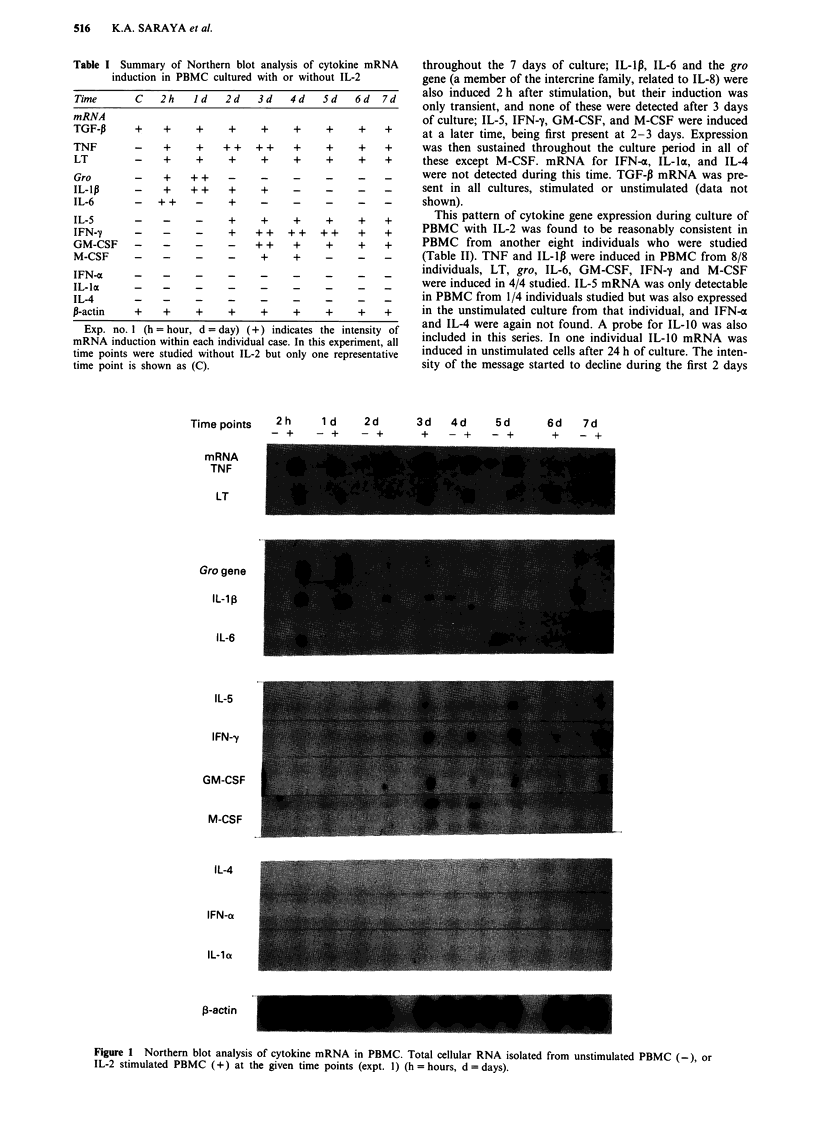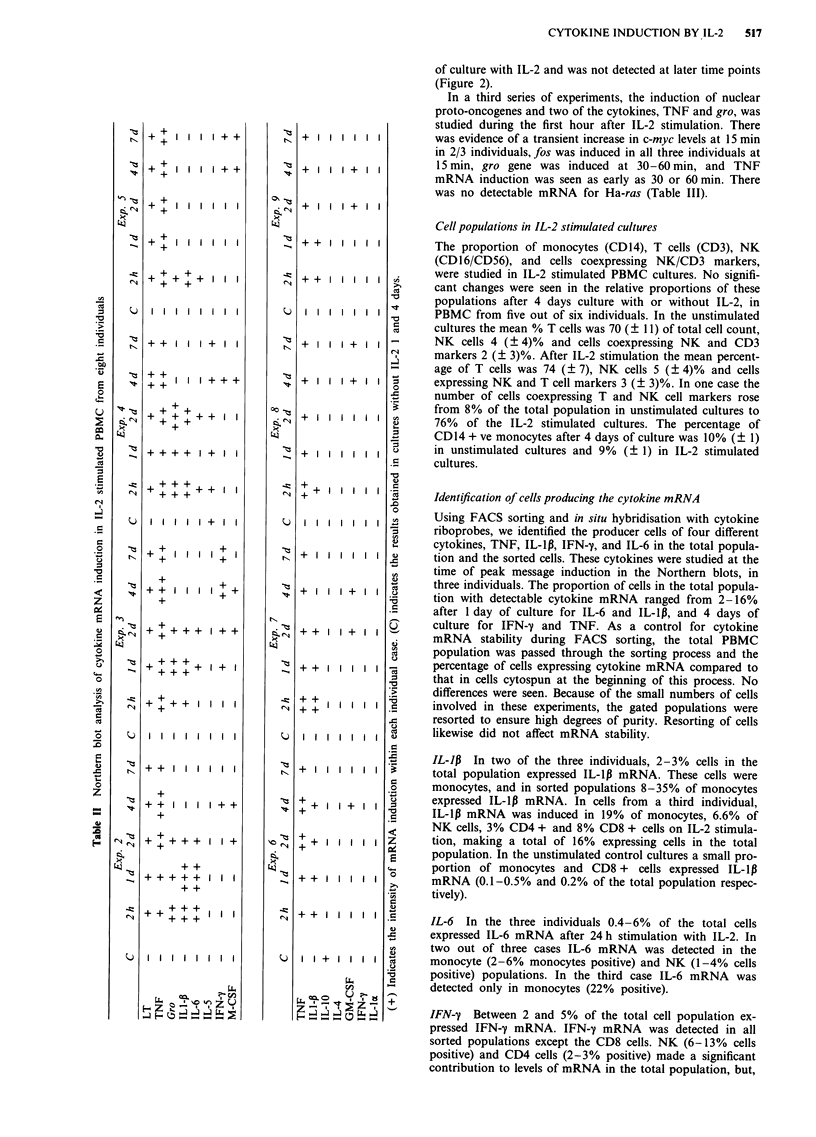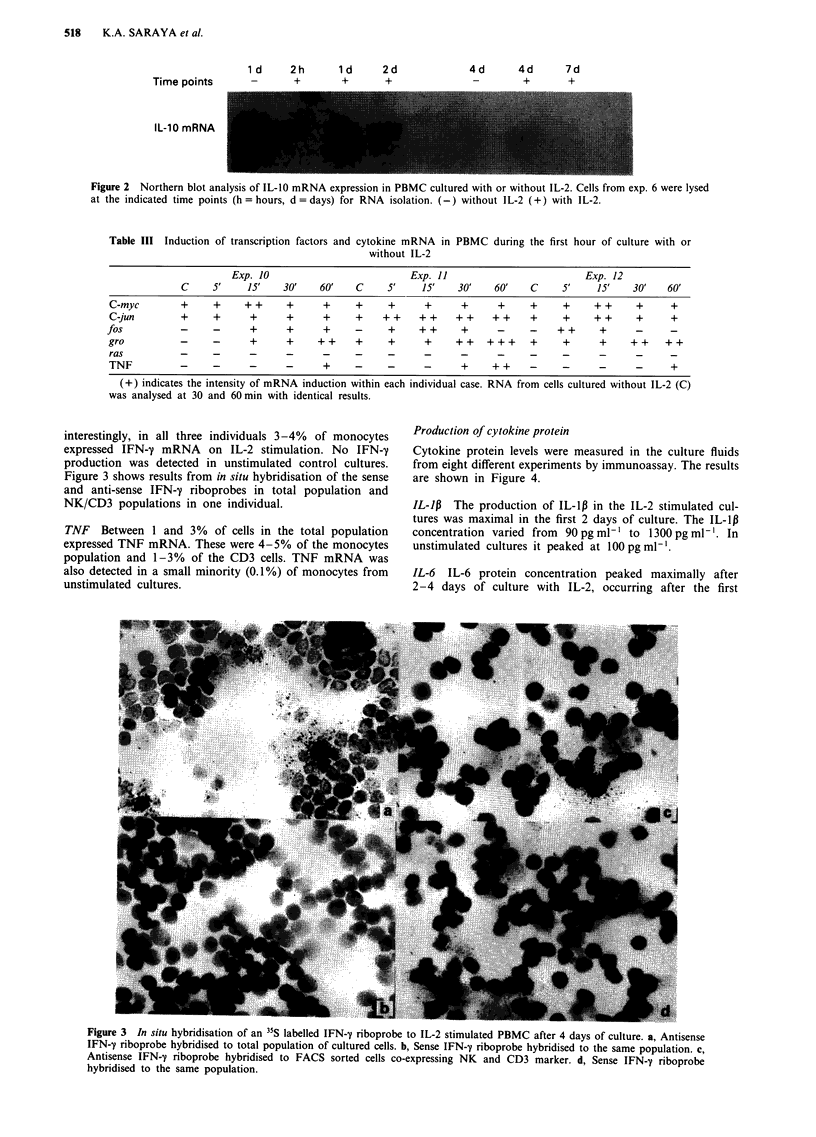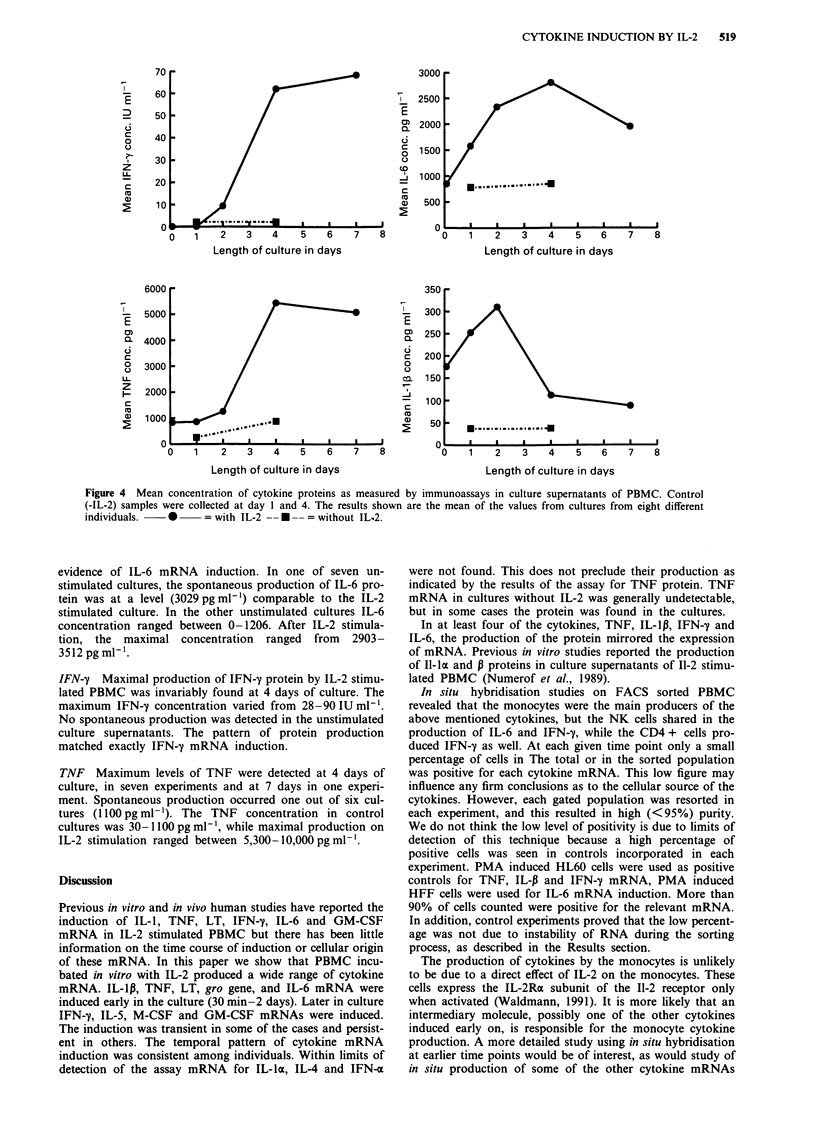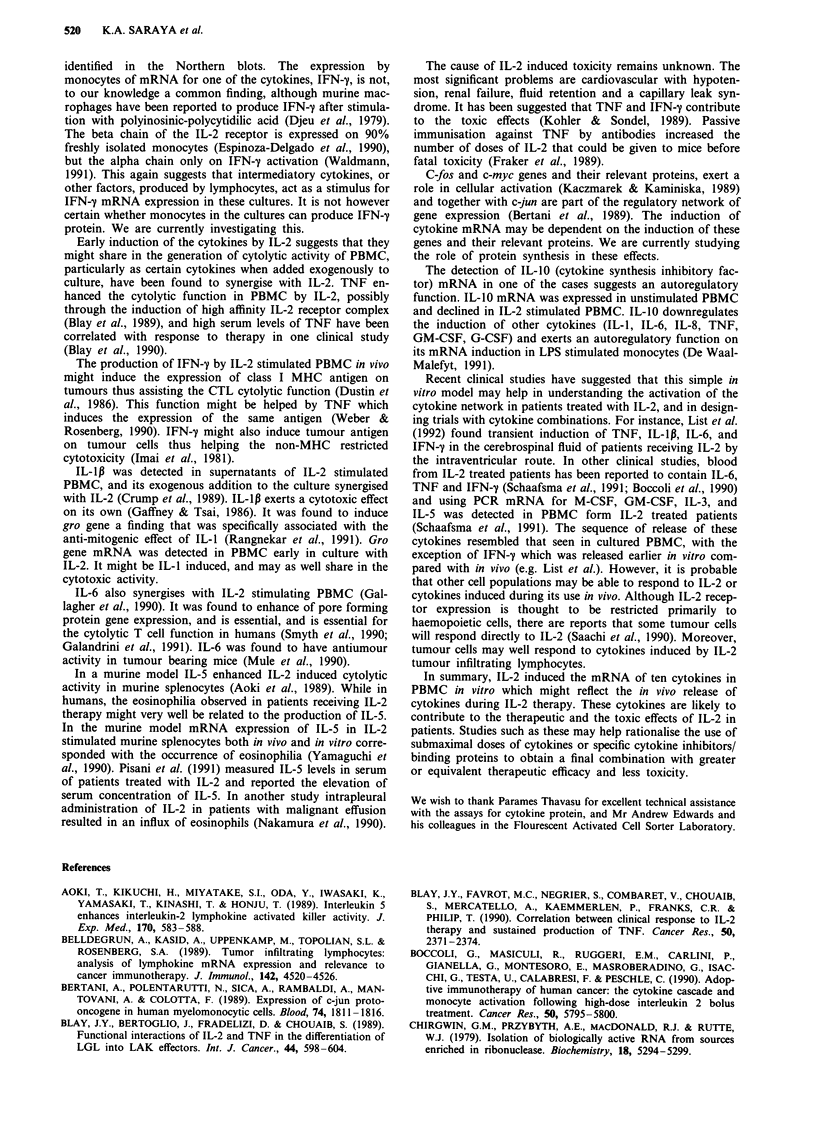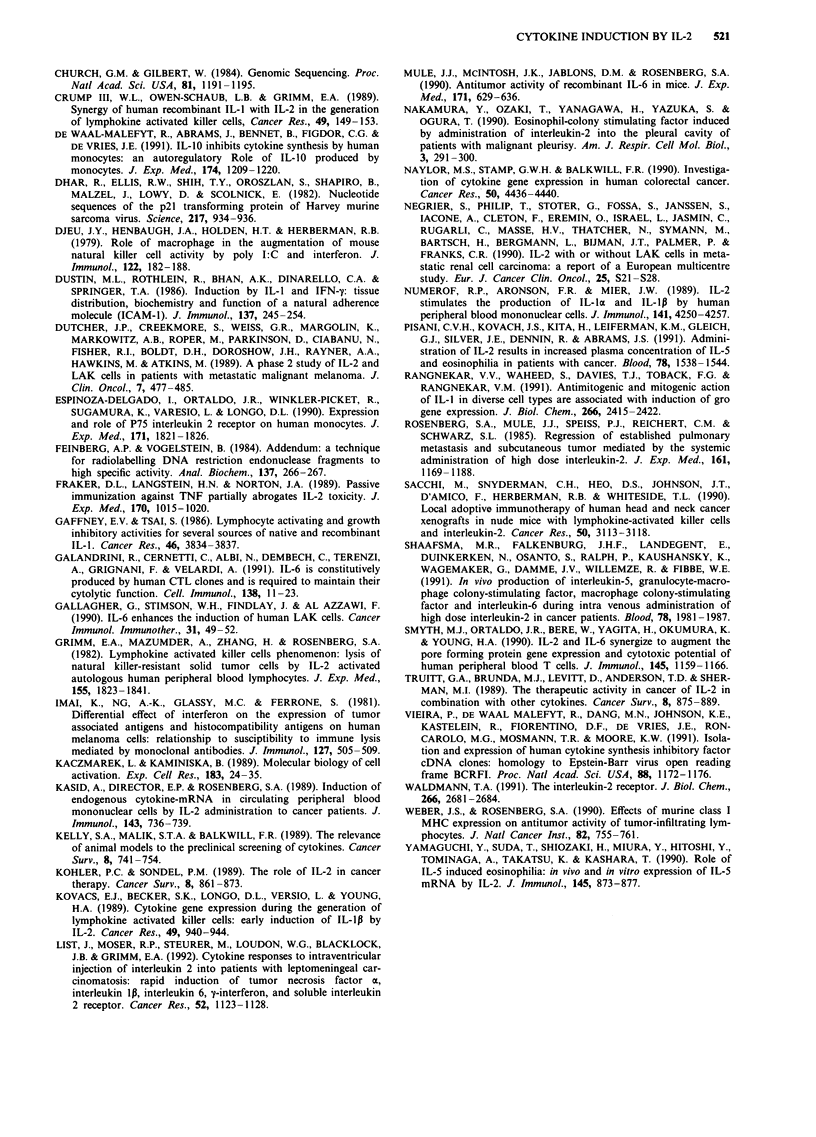# Temporal sequence and cellular origin of interleukin-2 stimulated cytokine gene expression.

**DOI:** 10.1038/bjc.1993.96

**Published:** 1993-03

**Authors:** K. A. Saraya, F. R. Balkwill

**Affiliations:** Biological Therapy Laboratory, Imperial Cancer Research Fund, Lincoln's Inn Fields, UK.

## Abstract

**Images:**


*Br. J. Cancer* (1993), **67**, 514–521 © Macmillan Press Ltd., 1993

### Temporal sequence and cellular origin of interleukin-2 stimulated cytokine gene expression

K.A. Saraya & F.R. Balkwill

*Biological Therapy Laboratory, Imperial Cancer Research Fund, Lincoln's Inn Fields, London WC2A 3PX, UK.*

**Summary** A study of activation of the cytokine network by interleukin 2, IL-2, may provide a rationale for devising cytokine combination and cytokine antagonist treatments with increased anti-tumour efficacy and decreased toxicity. We have investigated the expression of mRNA for 13 cytokines and three transcription factors during *in vitro* culture of peripheral blood mononuclear cells, PBMC, with IL-2. A consistent pattern of induction was seen in nine individuals, with early (2–24 h) induction of IL-1β, IL-6, tumour necrosis factor, TNF, lymphotoxin, LT, and *gro*. TNF and LT mRNA was expressed continually throughout culture, but levels of mRNA for IL-1β, IL-6, and *gro* declined by 24–48 h. After 48 h, PBMC began to express mRNA for IFN-γ, IL-5, GM-CSF, and M-CSF. At 15 min to 1 h post IL-2 mRNA for c-*fos*, c-*jun*, and c-*myc*, and TNF was induced in three individuals studied. IL-4, IFN-α, and IL-1α mRNA was not detected. Only a minority of cells expressed mRNA for TNF, IL-1β, IL-6 and IFN-γ, and monocytes were the main source. Levels of cytokine protein in culture supernatants mirrored the pattern of mRNA induction. This *in vitro* model shows clear parallels with the reported *in vivo* production of cytokines during IL-2 therapy, and may prove useful in designing new therapeutic strategies.

Therapy with IL-2, with or without *ex vivo* activated lymphocytes, has resulted in partial, and occasionally complete, tumour regressions in a minority of patients with melanoma and renal cell carcinoma (Dutcher *et al.*, 1989; Negrier *et al.*, 1990). The induction of cytolytic activity in peripheral blood mononuclear cells, PBMCs, by IL-2 has been well documented both *in vitro* and *in vivo* (Grimm *et al.*, 1982; Rosenberg *et al.*, 1985). The mechanisms of activation of this heterogeneous population of cells are not fully elucidated. Several studies have shown that *in vitro* activation results in production of several cytokines. Early (24 h) induction of IL-1α and IL-1β mRNA has been reported, as has the induction of TNF, LT and IFN-γ mRNA (Kovacs *et al.*, 1989). In other studies mRNA for GM-CSF and IL-6 was detected in PBMC after 3 days of culture with IL-2 (Belldegrun *et al.*, 1989; Kasid *et al.*, 1989).

The study of cytokines induced by IL-2 *in vitro* and *in vivo* is important for several reasons. First, these secretory products may contribute to the antitumour effects of IL-2 *in vivo*. Cytokines such as TNF and IFN-γ have well documented antitumour activity in animal models (Kelly *et al.*, 1989), and synergy between IL-2 and other cytokines has been reported (Truitt *et al.*, 1989). Second, the toxicity of IL-2 therapy may be related to the cytokines released by activated PBMC. For instance, anti-TNF antibody abrogated some of the toxic side effects of IL-2 in tumour bearing mice (Fraker *et al.*, 1989), and antibodies to IL-5 abolished the IL-2 induced eosinophilia in mice (Yamaguchi *et al.*, 1990). Third, studies of cytokine induction in a mixed population of mononuclear cells also gives us indications of the range of cytokines which may be induced after other mitogenic or antigenic stimuli.

In order to understand the potential of other cytokines or their antagonists, for influencing the anti-tumour activity or toxicity of IL-2 in humans, we have studied in detail the range and temporal sequence of cytokine mRNA and proteins induced by IL-2, and the producer cells of those cytokines. In this paper we present data obtained in *in vitro* cultures of human PBMC with IL-2.

### Materials and methods

### *Reagents*

Human rIL-2 was generously supplied by Glaxo IBM (Geneva, Switzerland) (specific activity $3.1 \times 10^6$ U mg$^{-1}$), and Roussel-UCLAF Centre de Recherche (Romaine, France) (specific actitivity $10^6$ U mg$^{-1}$). Maximum cytotoxicity was induced when the cells were cultured with 1000 U mg$^{-1}$ of IL-2.

### *Preparation of PBMCs*

Peripheral blood mononuclear cells from healthy donors were separated from heparinised venous blood on Ficoll-Hypaque gradient (Lymphoprep TM, Nycomed Pharma AS, Oslo, Norway). The cells were resuspended at $1 \times 10^6$ ml$^{-1}$ in RPMI 1640 containing 10% heat inactivated foetal calf serum with or without 1000 U ml rhIL-2. Cellular cytotoxicity was assayed after 3 days of culture. For northern blot analysis cells were lysed with guanidine-isothiocyanate solution for RNA extraction. For *in situ* hybridisation cells were immunostained for FACS sorting.

### *Cell lines as positive controls for Northern blot analysis*

The HL60 human promyelocytic leukaemic cell line was maintained in 5% FCS/RPMI 1640. Total cellular RNA was extracted after a 3 h incubation with 50 ng ml$^{-1}$ PMA (Sigma, Dorset, UK). This constituted a source of TNF, IL-1α, IL-1β, M-CSF, and TGF-β mRNA as a positive control for Northern blot analysis. Human T-cell line Jurkat cells were stimulated overnight with 1 ng ml$^{-1}$ phytohaemagglutinin (Sigma, Dorset, UK) and 20 ng ml$^{-1}$ PMA. Extracted RNA provided a positive control for IFN-γ. Human foreskin fibroblasts were stimulated with 10 ng ml$^{-1}$ PMA for 1 h, providing a positive control for IL-6 mRNA. MLA-144 T cell line stimulated with PHA and PMA provided a positive control for GM-CSF mRNA. It was not possible to obtain reliable controls for IFN-α and IL-4 expression using Northern analysis however the INF-α factor reacted on a Southern blot.

### *Labelling of target cells*

Freshly thawed T 24 cells were washed three times in complete medium. 0.1 mCi of $^{51}Cr$ ($Na_2{}^{51}CrO_4$) (Amersham International UK) was added to the cell pellet. Cells were incubated at 37°C for 1 h, then washed in complete medium three times to remove unbound $^{51}Cr$. Cells were resuspended in medium at a final concentration of $5 \times 10^4$ ml$^{-1}$.

### *Isolation of cellular RNA*

Non-adherent cells from PBMC cultures were harvested. The adherent cells were scraped with rubber policeman into

Correspondence: F.R. Balkwill.
Received 5 June 1992; and in revised form 2 October 1992.

guanidine-isothiocyanate solution which was then used to lyse the non-adherent fraction. Total cellular RNA was isolated after centrifugation through cesium chloride followed by precipitation with 3 M sodium acetate and ethanol (Chirgwin *et al.*, 1979).

*Northern blot analysis*

Fifteen μg aliquots of total cellular RNA were electrophoresed through a 1.4% agarose-formaldehyde denaturing gel then capillary blotted onto Biodyne A membrane (Pall Ultrafine Filteration Corp., Glen Cove, NY). Membrane were hybridised to $^{32}$P-labelled inserts of human complementary DNA probes under standard conditions as outlined by Church and Gilbert (1984) and labelled with [$^{32}$P]dCTP (Amersham International UK) by the random priming method of Feinberg and Vogelstein (1984). Membranes were subsequently washed to high stringency and exposed to Kodak XAR5 film at − 70°C with two intensifying screens (Dupont, Stevenage, Herts, United Kingdom). Films were exosed for 7 days.

In situ *hybridisation*

The technique used was essentially as described in Naylor *et al.* (1990) except that no prehybridisation stages were needed with cytospins. 10 μl of the hybridisation buffer containing $^{35}$S-labelled RNA probe at $5 \times 10^4$ dpm μl$^{-1}$, was added to each slide, which was then covered with a siliconised coverslip and left to hybridise at 50°C for 16 h. Slides were washed to high stringency at 50°C for 30 min, then 65°C for 16 h. Slides were washed to high stringency at 50°C for 30 min, then 65°C, followed by RNAse treatment to digest the unbound RNA followed by autoradiography. Slides were developed after 10 days of exposure and stained with methylene blue.

*Probes*

*For Northern analysis* The following probes were used: TNF: *Pst*I fragment of p-hTNF 1; IFN-γ: *Bam*HI fragment of pBR327g0-2; IL-1α: *Hind*III-*Eco*RI fragment of pSPhIL-1α.2; IL-1b: *Hind*III fragment of pSPhL-1β.2; IL-6: *Bgl*II fragment of pSP65T-IL-6; TGF-βαω *Eco*RI fragment pbas TGF-β1 (Genentech, California); β-actin: *Eco*RI-*Hind*III fragment of M13 β-actin; IL-5: *Xba*I-*Bam*HI fragment of pGEM4 IL-5; M-CSF: *Pst*I-*Sma*I fragment of pUC18 M-CSF; GM-CSF: *HXh*I fragment of pXM GM-CSF; *Gro*: gene*Eco*RI fragment of pGEM3 *Gro*; IFN-α: *Eco*RI fragment of PIF 211-hIFN-α2 (Wellcome, Beckenam, Kent, UK); LT- *Hind*III-*PVU*II fragment of pOKBLT-6-hLT; IL-10: cDNA clone (Vieira *et al.*, 1991). c-*myc*: *Bam*HI-*Sal*I fragment of DOR h-c-*myc*; c-*jun*: *Eco*RI-*Bam*HI fragment of M13 c-*jun*; *fos*: *Bgl*I-*Pvu*II fragment of pFBH-1-*fos*; *ras*: *Eco*RI fragment of pBR322 *ras*-Ha (Dhar *et al.*, 1982).

*For* in situ *hybridisation* An antisense β-actin riboprobe was generated from *Hind*III cleaved Bluescript M13 β-actin using T7 RNA polymerase. This was used as a positive controls in all runs. Antisense TNF was generated from the *Apa*I cleaved pGEM1-hTNF using T7 RNA polymerase (Promega Biotech, Madison WI). The negative control was sense TNF generated from *Bam*HI cleaved pGEM-hTNF using Sp6 RNA polymerase (Promega, Biotech). The antisense IFN-γ riboprobe was generated from *Apa*I cleaved pGEM-3 using T7 polymerase, the sense control was generated from *Eco*RI cleaved pGEM-3 using Sp6 polymerase. Antisense IL-1β riboprobe was generated from *Eco*RI linearised pSP h IL-1-β.2 Antisense IL-6 riboprobe was generated from *Acc*I linearised pGEM-3 using T7 polymerase, sense control from *Xba*I linearised pGEM-3 using Sp6 polymerase. *In vitro* transcriptions were performed using Promega Biotech transcription kits to incorporate $^{35}$S-UTP (Amersham International plc, UK). Restriction enzymes were obtained from Pharmacia.

*Flow cytometry and cell sorting*

On days 1 and 4 of culture in the presence or absence of IL-2, aliquots of PMBC were stained by direct immunofluorescence following the standard procedure recommended by the supplier of the fluorescent conjugates (Becton Dickinson Immunocytometry Systems, San Jose, Ca, USA). Non adherent and adherent cell populations were pooled for the analysis, adherent cells being removed from the culture by rubber policeman after incubation on ice for 1 h. Cells were labelled with either (a) anti-Leu-M3/phycoerythrin (PE) (CD-14), (b) Simultest anti-Leu-4/FITC (CD3) + anti-Leu-11C/PE (CD16) + anti-Leu-19/PE (CD56) or (c) Simultest anti-Leu-3a/FITC (CD4) + anti-Leu-2a/PE (CD8). All samples were examined using a FACStar$^{PLUS}$ (Becton Dickinson Immunocytometry Systems) and relevant populations sorted. The sorted populations were then recycled through the FACStar$^{PLUS}$ to ensure a high degree of purity. The flow cytometer was flushed with 0.1% diethylpyrocarbonate in phosphate buffered saline for 2 h prior to sorting. The collected cells were centrifuged at 1000 r.p.m. for 5 min at 4°C resuspended at a concentration of $1 \times 10^6$ cells ml$^{-1}$.

*Preparation of slides*

Sorted cells were then spun onto slides using a Shandon Cytospin centrifuge (Shandon Scientific Ltd., Astmoor, Runcoon, UK) at 500 r.p.m. × 5 min. Slides were then fixed in 4% paraformaldehyde (Sigma, Dorset, UK) in phosphate buffered saline, alcohol dehydrated and stored at − 70°C.

*Cytokine protein assays*

IL-1β was measured using EASIA kit from Medgenix (Brussels, Belgium) with a range of 33–1400 pg ml$^{-1}$. It was standardised against an international reference preparation (86/680) from the National Institute of Biological Standards and Controls, NIBSC, and was used at detection limits of 33 pg ml$^{-1}$. TNF-α and IFN-γ were measured using IRMA kits from Medgenix (Brussels, Belgium). The TNF-α IRMA range was from 15–5000 pg ml$^{-1}$. It was standardised against an international reference preparation (87/650) from the National Institute of Biological Standards and Controls, NIBSC, and was used at detection limits of 30 pg ml$^{-1}$. The IFN-γ-IRMA range was from 0–90 U ml$^{-1}$. It was standardised against an international reference preparation (88/606) from NIBSC and was used at detection limits of 2.5 U ml$^{-1}$. The IFN-α was measured using RIA kits from Medgenix (Brussels, Belgium). The range was from 0–200 U ml$^{-1}$. It was standardised against an international reference preparation (82/576) from NIBSC and was used at detection limits of 8.0 U ml$^{-1}$. IL-6 was an 'in house' RIA (antisera G150 BM from Dr S. Poole, NIBSC). The antisera was used at a final dilution of 1:1,750,000 (equivalent to an initial dilution of 1:350,000). The assay was standardised against an international reference preparation (88/514) from NIBSC and was used at detection limits of 70 pg ml$^{-1}$.

### Results

*Induction of cytokine and proto-oncogene mRNA in IL-2 stimulated cultures-Northern analysis*

In the first series of experiments we studied the induction of mRNA for 13 different cytokines in PBMC stimulated with 10,000 U ml$^{-1}$ IL-2, a concentration of IL-2 that generated maximal *in vitro* cytotoxicity against T24 cells. Table I and Figure 1 show the results obtained in cultures from one normal individual. In the interests of clarity, Table I only shows the mRNA induction in IL-2 stimulated cultures. The figure, however, clearly shows that there was no appreciable induction of cytokine mRNA in the cultures without IL-2. In this first individual, there were three different patterns of cytokine induction: TNF and LT were detected as early as 2 h after IL-2 addition and the mRNA was then present

**Table I** Summary of Northern blot analysis of cytokine mRNA induction in PBMC cultured with or without IL-2

| *Time* | *C* | *2 h* | *1 d* | *2 d* | *3 d* | *4 d* | *5 d* | *6 d* | *7 d* |
|---|---|---|---|---|---|---|---|---|---|
| *mRNA* | | | | | | | | | |
| TGF-β | + | + | + | + | + | + | + | + | + |
| TNF | – | + | + | ++ | ++ | + | + | + | + |
| LT | – | + | + | + | + | + | + | + | + |
| *Gro* | – | + | ++ | – | – | – | – | – | – |
| IL-1β | – | + | ++ | + | + | – | – | – | – |
| IL-6 | – | ++ | – | + | – | – | – | – | – |
| IL-5 | – | – | – | + | + | + | + | + | + |
| IFN-γ | – | – | – | + | ++ | ++ | ++ | + | + |
| GM-CSF | – | – | – | – | ++ | + | + | + | + |
| M-CSF | – | – | – | – | + | + | – | – | – |
| IFN-α | – | – | – | – | – | – | – | – | – |
| IL-1α | – | – | – | – | – | – | – | – | – |
| IL-4 | – | – | – | – | – | – | – | – | – |
| β-actin | + | + | + | + | + | + | + | + | + |

Exp. no. 1 (h = hour, d = day) (+) indicates the intensity of mRNA induction within each individual case. In this experiment, all time points were studied without IL-2 but only one representative time point is shown as (C).

throughout the 7 days of culture; IL-1β, IL-6 and the *gro* gene (a member of the intercrine family, related to IL-8) were also induced 2 h after stimulation, but their induction was only transient, and none of these were detected after 3 days of culture; IL-5, IFN-γ, GM-CSF, and M-CSF were induced at a later time, being first present at 2–3 days. Expression was then sustained throughout the culture period in all of these except M-CSF. mRNA for IFN-α, IL-1α, and IL-4 were not detected during this time. TGF-β mRNA was present in all cultures, stimulated or unstimulated (data not shown).

This pattern of cytokine gene expression during culture of PBMC with IL-2 was found to be reasonably consistent in PBMC from another eight individuals who were studied (Table II). TNF and IL-1β were induced in PBMC from 8/8 individuals, LT, *gro*, IL-6, GM-CSF, IFN-γ and M-CSF were induced in 4/4 studied. IL-5 mRNA was only detectable in PBMC from 1/4 individuals studied but was also expressed in the unstimulated culture from that individual, and IFN-α and IL-4 were again not found. A probe for IL-10 was also included in this series. In one individual IL-10 mRNA was induced in unstimulated cells after 24 h of culture. The intensity of the message started to decline during the first 2 days

**Figure 1** Northern blot analysis of cytokine mRNA in PBMC. Total cellular RNA isolated from unstimulated PBMC (–), or IL-2 stimulated PBMC (+) at the given time points (expt. 1) (h = hours, d = days).

**Table II** Northern blot analysis of cytokine mRNA induction in IL-2 stimulated PBMC from eight individuals

| | *Exp. 2* | | | | | | *Exp. 3* | | | | | | *Exp. 4* | | | | | | *Exp. 5* | | | | | |
|---|---|---|---|---|---|---|---|---|---|---|---|---|---|---|---|---|---|---|---|---|---|---|---|---|
| | *C* | *2 h* | *1 d* | *2 d* | *4 d* | *7 d* | *C* | *2 h* | *1 d* | *2 d* | *4 d* | *7 d* | *C* | *2 h* | *1 d* | *2 d* | *4 d* | *7 d* | *C* | *2 h* | *1 d* | *2 d* | *4 d* | *7 d* |
| LT | – | + | + | + | + | + | – | + | + | + | + | + | – | + | + | + | ++ | + | – | + | + | + | + | + |
| TNF | – | + | + | ++ | +++ | + | – | ++ | ++ | ++ | +++ | ++ | – | ++ | + | ++ | ++ | + | – | ++ | ++ | ++ | ++ | ++ |
| *Gro* | – | ++ | + | + | – | – | – | + | ++ | + | – | – | – | ++ | + | +++ | – | – | – | + | – | – | – | – |
| IL1-β | – | ++ | +++ | + | – | – | – | + | ++ | + | – | – | – | ++ | + | ++ | – | – | – | ++ | – | – | – | – |
| IL-6 | – | ++ | +++ | + | – | – | – | – | + | + | – | – | – | + | – | + | – | – | – | + | – | – | – | – |
| IL-5 | – | – | – | – | – | – | – | – | – | – | – | – | + | + | + | + | + | + | – | – | – | – | – | – |
| IFN-γ | – | – | – | – | + | – | – | – | + | + | ++ | ++ | – | – | – | – | + | – | – | – | – | – | + | + |
| M-CSF | – | – | – | + | + | – | – | – | – | + | + | – | – | – | – | – | + | – | – | – | – | – | + | + |

| | *Exp. 6* | | | | | | *Exp. 7* | | | | | | *Exp. 8* | | | | | | *Exp. 9* | | | | | |
|---|---|---|---|---|---|---|---|---|---|---|---|---|---|---|---|---|---|---|---|---|---|---|---|---|
| | *C* | *2 h* | *1 d* | *2 d* | *4 d* | *7 d* | *C* | *2 h* | *1 d* | *2 d* | *4 d* | *7 d* | *C* | *2 h* | *1 d* | *2 d* | *4 d* | *7 d* | *C* | *2 h* | *1 d* | *2 d* | *4 d* | *7 d* |
| TNF | – | + | + | ++ | ++ | + | – | ++ | + | + | + | + | – | ++ | + | + | + | + | – | + | + | + | + | + |
| IL1-β | – | + | + | + | + | – | – | ++ | + | + | – | – | – | + | – | – | – | – | – | + | + | – | – | – |
| IL-10 | + | – | – | – | – | – | – | – | – | – | – | – | – | – | – | – | – | – | – | – | – | – | – | – |
| IL-4 | – | – | – | – | – | – | – | – | – | – | – | – | – | – | – | – | – | – | – | – | – | – | – | – |
| GM-CSF | – | – | – | – | + | – | – | – | – | + | + | – | – | – | – | – | + | + | – | – | – | + | + | – |
| IFN-γ | – | – | – | – | – | – | – | – | – | – | – | – | – | – | – | – | – | – | – | – | – | – | – | – |
| IL-1α | – | – | – | – | – | – | – | – | – | – | – | – | – | – | – | – | – | – | – | – | – | – | – | – |

(+) Indicates the intensity of mRNA induction within each individual case. (C) indicates the results obtained in cultures without IL-2 1 and 4 days.

of culture with IL-2 and was not detected at later time points (Figure 2).

In a third series of experiments, the induction of nuclear proto-oncogenes and two of the cytokines, TNF and *gro*, was studied during the first hour after IL-2 stimulation. There was evidence of a transient increase in c-*myc* levels at 15 min in 2/3 individuals, *fos* was induced in all three individuals at 15 min, *gro* gene was induced at 30–60 min, and TNF mRNA induction was seen as early as 30 or 60 min. There was no detectable mRNA for Ha-*ras* (Table III).

### *Cell populations in IL-2 stimulated cultures*

The proportion of monocytes (CD14), T cells (CD3), NK (CD16/CD56), and cells coexpressing NK/CD3 markers, were studied in IL-2 stimulated PBMC cultures. No significant changes were seen in the relative proportions of these populations after 4 days culture with or without IL-2, in PBMC from five out of six individuals. In the unstimulated cultures the mean % T cells was 70 (± 11) of total cell count, NK cells 4 (± 4)% and cells coexpressing NK and CD3 markers 2 (± 3)%. After IL-2 stimulation the mean percentage of T cells was 74 (± 7), NK cells 5 (± 4)% and cells expressing NK and T cell markers 3 (± 3)%. In one case the number of cells coexpressing T and NK cell markers rose from 8% of the total population in unstimulated cultures to 76% of the IL-2 stimulated cultures. The percentage of CD14 + ve monocytes after 4 days of culture was 10% (± 1) in unstimulated cultures and 9% (± 1) in IL-2 stimulated cultures.

### *Identification of cells producing the cytokine mRNA*

Using FACS sorting and *in situ* hybridisation with cytokine riboprobes, we identified the producer cells of four different cytokines, TNF, IL-1β, IFN-γ, and IL-6 in the total population and the sorted cells. These cytokines were studied at the time of peak message induction in the Northern blots, in three individuals. The proportion of cells in the total population with detectable cytokine mRNA ranged from 2–16% after 1 day of culture for IL-6 and IL-1β, and 4 days of culture for IFN-γ and TNF. As a control for cytokine mRNA stability during FACS sorting, the total PBMC population was passed through the sorting process and the percentage of cells expressing cytokine mRNA compared to that in cells cytospun at the beginning of this process. No differences were seen. Because of the small numbers of cells involved in these experiments, the gated populations were resorted to ensure high degrees of purity. Resorting of cells likewise did not affect mRNA stability.

*IL-1β* In two of the three individuals, 2–3% cells in the total population expressed IL-1β mRNA. These cells were monocytes, and in sorted populations 8–35% of monocytes expressed IL-1β mRNA. In cells from a third individual, IL-1β mRNA was induced in 19% of monocytes, 6.6% of NK cells, 3% CD4 + and 8% CD8 + cells on IL-2 stimulation, making a total of 16% expressing cells in the total population. In the unstimulated control cultures a small proportion of monocytes and CD8 + cells expressed IL-1β mRNA (0.1–0.5% and 0.2% of the total population respectively).

*IL-6* In the three individuals 0.4–6% of the total cells expressed IL-6 mRNA after 24 h stimulation with IL-2. In two out of three cases IL-6 mRNA was detected in the monocyte (2–6% monocytes positive) and NK (1–4% cells positive) populations. In the third case IL-6 mRNA was detected only in monocytes (22% positive).

*IFN-γ* Between 2 and 5% of the total cell population expressed IFN-γ mRNA. IFN-γ mRNA was detected in all sorted populations except the CD8 cells. NK (6–13% cells positive) and CD4 cells (2–3% positive) made a significant contribution to levels of mRNA in the total population, but,

**Figure 2** Northern blot analysis of IL-10 mRNA expression in PBMC cultured with or without IL-2. Cells from exp. 6 were lysed at the indicated time points (h = hours, d = days) for RNA isolation. (−) without IL-2 (+) with IL-2.

**Table III** Induction of transcription factors and cytokine mRNA in PBMC during the first hour of culture with or without IL-2

| | *Exp. 10* | | | | | *Exp. 11* | | | | | *Exp. 12* | | | | |
|---|---|---|---|---|---|---|---|---|---|---|---|---|---|---|---|
| | *C* | *5′* | *15′* | *30′* | *60′* | *C* | *5′* | *15′* | *30′* | *60′* | *C* | *5′* | *15′* | *30′* | *60′* |
| C-*myc* | + | + | ++ | + | + | + | + | + | + | + | + | + | ++ | + | + |
| C-*jun* | + | + | + | + | + | + | ++ | ++ | ++ | ++ | + | + | ++ | + | + |
| *fos* | − | − | + | + | + | − | + | ++ | + | − | − | ++ | + | − | − |
| *gro* | − | − | + | + | ++ | + | + | + | ++ | +++ | + | + | + | ++ | ++ |
| *ras* | − | − | − | − | − | − | − | − | − | − | − | − | − | − | − |
| TNF | − | − | − | − | + | − | − | − | + | ++ | − | − | − | − | + |

(+) indicates the intensity of mRNA induction within each individual case. RNA from cells cultured without IL-2 (C) was analysed at 30 and 60 min with identical results.

interestingly, in all three individuals 3–4% of monocytes expressed IFN-γ mRNA on IL-2 stimulation. No IFN-γ production was detected in unstimulated control cultures. Figure 3 shows results from *in situ* hybridisation of the sense and anti-sense IFN-γ riboprobes in total population and NK/CD3 populations in one individual.

*TNF* Between 1 and 3% of cells in the total population expressed TNF mRNA. These were 4–5% of the monocytes population and 1–3% of the CD3 cells. TNF mRNA was also detected in a small minority (0.1%) of monocytes from unstimulated cultures.

*Production of cytokine protein*

Cytokine protein levels were measured in the culture fluids from eight different experiments by immunoassay. The results are shown in Figure 4.

*IL-1β* The production of IL-1β in the IL-2 stimulated cultures was maximal in the first 2 days of culture. The IL-1β concentration varied from 90 pg $ml^{-1}$ to 1300 pg $ml^{-1}$. In unstimulated cultures it peaked at 100 pg $ml^{-1}$.

*IL-6* IL-6 protein concentration peaked maximally after 2–4 days of culture with IL-2, occurring after the first

**Figure 3** *In situ* hybridisation of an $^{35}S$ labelled IFN-γ riboprobe to IL-2 stimulated PBMC after 4 days of culture. **a**, Antisense IFN-γ riboprobe hybridised to total population of cultured cells. **b**, Sense IFN-γ riboprobe hybridised to the same population. **c**, Antisense IFN-γ riboprobe hybridised to FACS sorted cells co-expressing NK and CD3 marker. **d**, Sense IFN-γ riboprobe hybridised to the same population.

**Figure 4** Mean concentration of cytokine proteins as measured by immunoassays in culture supernatants of PBMC. Control (-IL-2) samples were collected at day 1 and 4. The results shown are the mean of the values from cultures from eight different individuals. ——●—— = with IL-2 --■-- = without IL-2.

evidence of IL-6 mRNA induction. In one of seven unstimulated cultures, the spontaneous production of IL-6 protein was at a level (3029 pg $ml^{-1}$) comparable to the IL-2 stimulated culture. In the other unstimulated cultures IL-6 concentration ranged between 0–1206. After IL-2 stimulation, the maximal concentration ranged from 2903–3512 pg $ml^{-1}$.

*IFN-γ* Maximal production of IFN-γ protein by IL-2 stimulated PBMC was invariably found at 4 days of culture. The maximum IFN-γ concentration varied from 28–90 IU $ml^{-1}$. No spontaneous production was detected in the unstimulated culture supernatants. The pattern of protein production matched exactly IFN-γ mRNA induction.

*TNF* Maximum levels of TNF were detected at 4 days of culture, in seven experiments and at 7 days in one experiment. Spontaneous production occurred one out of six cultures (1100 pg $ml^{-1}$). The TNF concentration in control cultures was 30–1100 pg $ml^{-1}$, while maximal production on IL-2 stimulation ranged between 5,300–10,000 pg $ml^{-1}$.

### Discussion

Previous *in vitro* and *in vivo* human studies have reported the induction of IL-1, TNF, LT, IFN-γ, IL-6 and GM-CSF mRNA in IL-2 stimulated PBMC but there has been little information on the time course of induction or cellular origin of these mRNA. In this paper we show that PBMC incubated *in vitro* with IL-2 produced a wide range of cytokine mRNA. IL-1β, TNF, LT, *gro* gene, and IL-6 mRNA were induced early in the culture (30 min–2 days). Later in culture IFN-γ, IL-5, M-CSF and GM-CSF mRNAs were induced. The induction was transient in some of the cases and persistent in others. The temporal pattern of cytokine mRNA induction was consistent among individuals. Within limits of detection of the assay mRNA for IL-1α, IL-4 and IFN-α were not found. This does not preclude their production as indicated by the results of the assay for TNF protein. TNF mRNA in cultures without IL-2 was generally undetectable, but in some cases the protein was found in the cultures.

In at least four of the cytokines, TNF, IL-1β, IFN-γ and IL-6, the production of the protein mirrored the expression of mRNA. Previous *in vitro* studies reported the production of Il-1α and β proteins in culture supernatants of Il-2 stimulated PBMC (Numerof *et al.*, 1989).

*In situ* hybridisation studies on FACS sorted PBMC revealed that the monocytes were the main producers of the above mentioned cytokines, but the NK cells shared in the production of IL-6 and IFN-γ, while the CD4 + cells produced IFN-γ as well. At each given time point only a small percentage of cells in The total or in the sorted population was positive for each cytokine mRNA. This low figure may influence any firm conclusions as to the cellular source of the cytokines. However, each gated population was resorted in each experiment, and this resulted in high (<95%) purity. We do not think the low level of positivity is due to limits of detection of this technique because a high percentage of positive cells was seen in controls incorporated in each experiment. PMA induced HL60 cells were used as positive controls for TNF, IL-β and IFN-γ mRNA, PMA induced HFF cells were used for IL-6 mRNA induction. More than 90% of cells counted were positive for the relevant mRNA. In addition, control experiments proved that the low percentage was not due to instability of RNA during the sorting process, as described in the Results section.

The production of cytokines by the monocytes is unlikely to be due to a direct effect of IL-2 on the monocytes. These cells express the IL-2Rα subunit of the Il-2 receptor only when activated (Waldmann, 1991). It is more likely that an intermediary molecule, possibly one of the other cytokines induced early on, is responsible for the monocyte cytokine production. A more detailed study using *in situ* hybridisation at earlier time points would be of interest, as would study of *in situ* production of some of the other cytokine mRNAs identified in the Northern blots. The expression by monocytes of mRNA for one of the cytokines, IFN-γ, is not, to our knowledge a common finding, although murine macrophages have been reported to produce IFN-γ after stimulation with polyinosinic-polycytidilic acid (Djeu *et al.*, 1979). The beta chain of the IL-2 receptor is expressed on 90% freshly isolated monocytes (Espinoza-Delgado *et al.*, 1990), but the alpha chain only on IFN-γ activation (Waldmann, 1991). This again suggests that intermediatory cytokines, or other factors, produced by lymphocytes, act as a stimulus for IFN-γ mRNA expression in these cultures. It is not however certain whether monocytes in the cultures can produce IFN-γ protein. We are currently investigating this.

Early induction of the cytokines by IL-2 suggests that they might share in the generation of cytolytic activity of PBMC, particularly as certain cytokines when added exogenously to culture, have been found to synergise with IL-2. TNF enhanced the cytolytic function in PBMC by IL-2, possibly through the induction of high affinity IL-2 receptor complex (Blay *et al.*, 1989), and high serum levels of TNF have been correlated with response to therapy in one clinical study (Blay *et al.*, 1990).

The production of IFN-γ by IL-2 stimulated PBMC *in vivo* might induce the expression of class I MHC antigen on tumours thus assisting the CTL cytolytic function (Dustin *et al.*, 1986). This function might be helped by TNF which induces the expression of the same antigen (Weber & Rosenberg, 1990). IFN-γ might also induce tumour antigen on tumour cells thus helping the non-MHC restricted cytotoxicity (Imai *et al.*, 1981).

IL-1β was detected in supernatants of IL-2 stimulated PBMC, and its exogenous addition to the culture synergised with IL-2 (Crump *et al.*, 1989). IL-1β exerts a cytotoxic effect on its own (Gaffney & Tsai, 1986). It was found to induce *gro* gene a finding that was specifically associated with the anti-mitogenic effect of IL-1 (Rangnekar *et al.*, 1991). *Gro* gene mRNA was detected in PBMC early in culture with IL-2. It might be IL-1 induced, and may as well share in the cytotoxic activity.

IL-6 also synergises with IL-2 stimulating PBMC (Gallagher *et al.*, 1990). It was found to enhance of pore forming protein gene expression, and is essential, and is essential for the cytolytic T cell function in humans (Smyth *et al.*, 1990; Galandrini *et al.*, 1991). IL-6 was found to have antiumour activity in tumour bearing mice (Mule *et al.*, 1990).

In a murine model IL-5 enhanced IL-2 induced cytolytic activity in murine splenocytes (Aoki *et al.*, 1989). While in humans, the eosinophilia observed in patients receiving IL-2 therapy might very well be related to the production of IL-5. In the murine model mRNA expression of IL-5 in IL-2 stimulated murine splenocytes both *in vivo* and *in vitro* corresponded with the occurrence of eosinophilia (Yamaguchi *et al.*, 1990). Pisani *et al.* (1991) measured IL-5 levels in serum of patients treated with IL-2 and reported the elevation of serum concentration of IL-5. In another study intrapleural administration of IL-2 in patients with malignant effusion resulted in an influx of eosinophils (Nakamura *et al.*, 1990).

The cause of IL-2 induced toxicity remains unknown. The most significant problems are cardiovascular with hypotension, renal failure, fluid retention and a capillary leak syndrome. It has been suggested that TNF and IFN-γ contribute to the toxic effects (Kohler & Sondel, 1989). Passive immunisation against TNF by antibodies increased the number of doses of IL-2 that could be given to mice before fatal toxicity (Fraker *et al.*, 1989).

*C-fos* and *c-myc* genes and their relevant proteins, exert a role in cellular activation (Kaczmarek & Kaminiska, 1989) and together with *c-jun* are part of the regulatory network of gene expression (Bertani *et al.*, 1989). The induction of cytokine mRNA may be dependent on the induction of these genes and their relevant proteins. We are currently studying the role of protein synthesis in these effects.

The detection of IL-10 (cytokine synthesis inhibitory factor) mRNA in one of the cases suggests an autoregulatory function. IL-10 mRNA was expressed in unstimulated PBMC and declined in IL-2 stimulated PBMC. IL-10 downregulates the induction of other cytokines (IL-1, IL-6, IL-8, TNF, GM-CSF, G-CSF) and exerts an autoregulatory function on its mRNA induction in LPS stimulated monocytes (De Waal-Malefyt, 1991).

Recent clinical studies have suggested that this simple *in vitro* model may help in understanding the activation of the cytokine network in patients treated with IL-2, and in designing trials with cytokine combinations. For instance, List *et al.* (1992) found transient induction of TNF, IL-1β, IL-6, and IFN-γ in the cerebrospinal fluid of patients receiving IL-2 by the intraventricular route. In other clinical studies, blood from IL-2 treated patients has been reported to contain IL-6, TNF and IFN-γ (Schaafsma *et al.*, 1991; Boccoli *et al.*, 1990) and using PCR mRNA for M-CSF, GM-CSF, IL-3, and IL-5 was detected in PBMC form IL-2 treated patients (Schaafsma *et al.*, 1991). The sequence of release of these cytokines resembled that seen in cultured PBMC, with the exception of IFN-γ which was released earlier *in vitro* compared with *in vivo* (e.g. List *et al.*). However, it is probable that other cell populations may be able to respond to IL-2 or cytokines induced during its use *in vivo*. Although IL-2 receptor expression is thought to be restricted primarily to haemopoietic cells, there are reports that some tumour cells will respond directly to IL-2 (Saachi *et al.*, 1990). Moreover, tumour cells may well respond to cytokines induced by IL-2 tumour infiltrating lymphocytes.

In summary, IL-2 induced the mRNA of ten cytokines in PBMC *in vitro* which might reflect the *in vivo* release of cytokines during IL-2 therapy. These cytokines are likely to contribute to the therapeutic and the toxic effects of IL-2 in patients. Studies such as these may help rationalise the use of submaximal doses of cytokines or specific cytokine inhibitors/binding proteins to obtain a final combination with greater or equivalent therapeutic efficacy and less toxicity.

We wish to thank Parames Thavasu for excellent technical assistance with the assays for cytokine protein, and Mr Andrew Edwards and his colleagues in the Flourescent Activated Cell Sorter Laboratory.

### References

AOKI, T., KIKUCHI, H., MIYATAKE, S.I., ODA, Y., IWASAKI, K., YAMASAKI, T., KINASHI, T. & HONJU, T. (1989). Interleukin 5 enhances interleukin-2 lymphokine activated killer activity. *J. Exp. Med.*, **170**, 583–588.

BELLDEGRUN, A., KASID, A., UPPENKAMP, M., TOPOLIAN, S.L. & ROSENBERG, S.A. (1989). Tumor infiltrating lymphocytes: analysis of lymphokine mRNA expression and relevance to cancer immunotherapy. *J. Immunol.*, **142**, 4520–4526.

BERTANI, A., POLENTARUTTI, N., SICA, A., RAMBALDI, A., MANTOVANI, A. & COLOTTA, F. (1989). Expression of c-jun protooncogene in human myelomonocytic cells. *Blood*, **74**, 1811–1816.

BLAY, J.Y., BERTOGLIO, J., FRADELIZI, D. & CHOUAIB, S. (1989). Functional interactions of IL-2 and TNF in the differentiation of LGL into LAK effectors. *Int. J. Cancer.*, **44**, 598–604.

BLAY, J.Y., FAVROT, M.C., NEGRIER, S., COMBARET, V., CHOUAIB, S., MERCATELLO, A., KAEMMERLEN, P., FRANKS, C.R. & PHILIP, T. (1990). Correlation between clinical response to IL-2 therapy and sustained production of TNF. *Cancer Res.*, **50**, 2371–2374.

BOCCOLI, G., MASICULI, R., RUGGERI, E.M., CARLINI, P., GIANELLA, G., MONTESORO, E., MASROBERADINO, G., ISACCHI, G., TESTA, U., CALABRESI, F. & PESCHLE, C. (1990). Adoptive immunotherapy of human cancer: the cytokine cascade and monocyte activation following high-dose interleukin 2 bolus treatment. *Cancer Res.*, **50**, 5795–5800.

CHIRGWIN, G.M., PRZYBYTH, A.E., MACDONALD, R.J. & RUTTE, W.J. (1979). Isolation of biologically active RNA from sources enriched in ribonuclease. *Biochemistry*, **18**, 5294–5299.

CHURCH, G.M. & GILBERT, W. (1984). Genomic Sequencing. *Proc. Natl Acad. Sci. USA*, **81**, 1191–1195.

CRUMP III, W.L., OWEN-SCHAUB, L.B. & GRIMM, E.A. (1989). Synergy of human recombinant IL-1 with IL-2 in the generation of lymphokine activated killer cells, *Cancer Res.*, **49**, 149–153.

DE WAAL-MALEFYT, R., ABRAMS, J., BENNET, B., FIGDOR, C.G. & DE VRIES, J.E. (1991). IL-10 inhibits cytokine synthesis by human monocytes: an autoregulatory Role of IL-10 produced by monocytes. *J. Exp. Med.*, **174**, 1209–1220.

DHAR, R., ELLIS, R.W., SHIH, T.Y., OROSZLAN, S., SHAPIRO, B., MALZEL, J., LOWY, D. & SCOLNICK, E. (1982). Nucleotide sequences of the p21 transforming protein of Harvey murine sarcoma virus. *Science*, **217**, 934–936.

DJEU, J.Y., HENBAUGH, J.A., HOLDEN, H.T. & HERBERMAN, R.B. (1979). Role of macrophage in the augmentation of mouse natural killer cell activity by poly I:C and interferon. *J. Immunol.*, **122**, 182–188.

DUSTIN, M.L., ROTHLEIN, R., BHAN, A.K., DINARELLO, C.A. & SPRINGER, T.A. (1986). Induction by IL-1 and IFN-γ: tissue distribution, biochemistry and function of a natural adherence molecule (ICAM-1). *J. Immunol.*, **137**, 245–254.

DUTCHER, J.P., CREEKMORE, S., WEISS, G.R., MARGOLIN, K., MARKOWITZ, A.B., ROPER, M., PARKINSON, D., CIABANU, N., FISHER, R.I., BOLDT, D.H., DOROSHOW, J.H., RAYNER, A.A., HAWKINS, M. & ATKINS, M. (1989). A phase 2 study of IL-2 and LAK cells in patients with metastatic malignant melanoma. *J. Clin. Oncol.*, **7**, 477–485.

ESPINOZA-DELGADO, I., ORTALDO, J.R., WINKLER-PICKET, R., SUGAMURA, K., VARESIO, L. & LONGO, D.L. (1990). Expression and role of P75 interleukin 2 receptor on human monocytes. *J. Exp. Med.*, **171**, 1821–1826.

FEINBERG, A.P. & VOGELSTEIN, B. (1984). Addendum: a technique for radiolabelling DNA restriction endonuclease fragments to high specific activity. *Anal. Biochem.*, **137**, 266–267.

FRAKER, D.L., LANGSTEIN, H.N. & NORTON, J.A. (1989). Passive immunization against TNF partially abrogates IL-2 toxicity. *J. Exp. Med.*, **170**, 1015–1020.

GAFFNEY, E.V. & TSAI, S. (1986). Lymphocyte activating and growth inhibitory activities for several sources of native and recombinant IL-1. *Cancer Res.*, **46**, 3834–3837.

GALANDRINI, R., CERNETTI, C., ALBI, N., DEMBECH, C., TERENZI, A., GRIGNANI, F. & VELARDI, A. (1991). IL-6 is constitutively produced by human CTL clones and is required to maintain their cytolytic function. *Cell. Immunol.*, **138**, 11–23.

GALLAGHER, G., STIMSON, W.H., FINDLAY, J. & AL AZZAWI, F. (1990). IL-6 enhances the induction of human LAK cells. *Cancer Immunol. Immunother.*, **31**, 49–52.

GRIMM, E.A., MAZUMDER, A., ZHANG, H. & ROSENBERG, S.A. (1982). Lymphokine activated killer cells phenomenon: lysis of natural killer-resistant solid tumor cells by IL-2 activated autologous human peripheral blood lymphocytes. *J. Exp. Med.*, **155**, 1823–1841.

IMAI, K., NG, A.-K., GLASSY, M.C. & FERRONE, S. (1981). Differential effect of interferon on the expression of tumor associated antigens and histocompatibility antigens on human melanoma cells: relationship to susciptibility to immune lysis mediated by monoclonal antibodies. *J. Immunol.*, **127**, 505–509.

KACZMAREK, L. & KAMINISKA, B. (1989). Molecular biology of cell activation. *Exp. Cell Res.*, **183**, 24–35.

KASID, A., DIRECTOR, E.P. & ROSENBERG, S.A. (1989). Induction of endogenous cytokine-mRNA in circulating peripheral blood mononuclear cells by IL-2 administration to cancer patients. *J. Immunol.*, **143**, 736–739.

KELLY, S.A., MALIK, S.T.A. & BALKWILL, F.R. (1989). The relevance of animal models to the preclinical screening of cytokines. *Cancer Surv.*, **8**, 741–754.

KOHLER, P.C. & SONDEL, P.M. (1989). The role of IL-2 in cancer therapy. *Cancer Surv.*, **8**, 861–873.

KOVACS, E.J., BECKER, S.K., LONGO, D.L., VERSIO, L. & YOUNG, H.A. (1989). Cytokine gene expression during the generation of lymphokine activated killer cells: early induction of IL-1β by IL-2. *Cancer Res.*, **49**, 940–944.

LIST, J., MOSER, R.P., STEURER, M., LOUDON, W.G., BLACKLOCK, J.B. & GRIMM, E.A. (1992). Cytokine responses to intraventricular injection of interleukin 2 into patients with leptomeningeal carcinomatosis: rapid induction of tumor necrosis factor α, interleukin 1β, interleukin 6, γ-interferon, and soluble interleukin 2 receptor. *Cancer Res.*, **52**, 1123–1128.

MULE, J.J., MCINTOSH, J.K., JABLONS, D.M. & ROSENBERG, S.A. (1990). Antitumor activity of recombinant IL-6 in mice. *J. Exp. Med.*, **171**, 629–636.

NAKAMURA, Y., OZAKI, T., YANAGAWA, H., YAZUKA, S. & OGURA, T. (1990). Eosinophil-colony stimulating factor induced by administration of interleukin-2 into the pleural cavity of patients with malignant pleurisy. *Am. J. Respir. Cell Mol. Biol.*, **3**, 291–300.

NAYLOR, M.S., STAMP, G.W.H. & BALKWILL, F.R. (1990). Investigation of cytokine gene expression in human colorectal cancer. *Cancer Res.*, **50**, 4436–4440.

NEGRIER, S., PHILIP, T., STOTER, G., FOSSA, S., JANSSEN, S., IACONE, A., CLETON, F., EREMIN, O., ISRAEL, L., JASMIN, C., RUGARLI, C., MASSE, H.V., THATCHER, N., SYMANN, M., BARTSCH, H., BERGMANN, L., BIJMAN, J.T., PALMER, P. & FRANKS, C.R. (1990). IL-2 with or without LAK cells in metastatic renal cell carcinoma: a report of a European multicentre study. *Eur. J. Cancer Clin. Oncol.*, **25**, S21–S28.

NUMEROF, R.P., ARONSON, F.R. & MIER, J.W. (1989). IL-2 stimulates the production of IL-1α and IL-1β by human peripheral blood mononuclear cells. *J. Immunol.*, **141**, 4250–4257.

PISANI, C.V.H., KOVACH, J.S., KITA, H., LEIFERMAN, K.M., GLEICH, G.J., SILVER, J.E., DENNIN, R. & ABRAMS, J.S. (1991). Administration of IL-2 results in increased plasma concentration of IL-5 and eosinophilia in patients with cancer. *Blood*, **78**, 1538–1544.

RANGNEKAR, V.V., WAHEED, S., DAVIES, T.J., TOBACK, F.G. & RANGNEKAR, V.M. (1991). Antimitogenic and mitogenic action of IL-1 in diverse cell types are associated with induction of gro gene expression. *J. Biol. Chem.*, **266**, 2415–2422.

ROSENBERG, S.A., MULE, J.J., SPEISS, P.J., REICHERT, C.M. & SCHWARZ, S.L. (1985). Regression of established pulmonary metastasis and subcutaneous tumor mediated by the systemic administration of high dose interleukin-2. *J. Exp. Med.*, **161**, 1169–1188.

SACCHI, M., SNYDERMAN, C.H., HEO, D.S., JOHNSON, J.T., D'AMICO, F., HERBERMAN, R.B. & WHITESIDE, T.L. (1990). Local adoptive immunotherapy of human head and neck cancer xenografts in nude mice with lymphokine-activated killer cells and interleukin-2. *Cancer Res.*, **50**, 3113–3118.

SHAAFSMA, M.R., FALKENBURG, J.H.F., LANDEGENT, E., DUINKERKEN, N., OSANTO, S., RALPH, P., KAUSHANSKY, K., WAGEMAKER, G., DAMME, J.V., WILLEMZE, R. & FIBBE, W.E. (1991). *In vivo* production of interleukin-5, granulocyte-macrophage colony-stimulating factor, macrophage colony-stimulating factor and interleukin-6 during intra venous administration of high dose interleukin-2 in cancer patients. *Blood*, **78**, 1981–1987.

SMYTH, M.J., ORTALDO, J.R., BERE, W., YAGITA, H., OKUMURA, K. & YOUNG, H.A. (1990). IL-2 and IL-6 synergize to augment the pore forming protein gene expression and cytotoxic potential of human peripheral blood T cells. *J. Immunol.*, **145**, 1159–1166.

TRUITT, G.A., BRUNDA, M.J., LEVITT, D., ANDERSON, T.D. & SHERMAN, M.I. (1989). The therapeutic activity in cancer of IL-2 in combination with other cytokines. *Cancer Surv.*, **8**, 875–889.

VIEIRA, P., DE WAAL MALEFYT, R., DANG, M.N., JOHNSON, K.E., KASTELEIN, R., FIORENTINO, D.F., DE VRIES, J.E., RONCAROLO, M.G., MOSMANN, T.R. & MOORE, K.W. (1991). Isolation and expression of human cytokine synthesis inhibitory factor cDNA clones: homology to Epstein-Barr virus open reading frame BCRFI. *Proc. Natl Acad. Sci. USA*, **88**, 1172–1176.

WALDMANN, T.A. (1991). The interleukin-2 receptor. *J. Biol. Chem.*, **266**, 2681–2684.

WEBER, J.S., & ROSENBERG, S.A. (1990). Effects of murine class I MHC expression on antitumor activity of tumor-infiltrating lymphocytes. *J. Natl Cancer Inst.*, **82**, 755–761.

YAMAGUCHI, Y., SUDA, T., SHIOZAKI, H., MIURA, Y., HITOSHI, Y., TOMINAGA, A., TAKATSU, K. & KASHARA, T. (1990). Role of IL-5 induced eosinophilia: *in vivo* and *in vitro* expression of IL-5 mRNA by IL-2. *J. Immunol.*, **145**, 873–877.